# Screening and treating problematic substance use among patients in psychiatry – obstacles and solutions

**DOI:** 10.1186/s13104-023-06389-w

**Published:** 2023-06-22

**Authors:** Elisabeth Petersén, Anne H Berman

**Affiliations:** 1 Centre for Psychiatry Research, Department of Clinical Neuroscience, Karolinska Institutet, Stockholm, Sweden; 2 Stockholm Health Care Services, Region Stockholm, Stockholm, Sweden; 3 Department of Psychology, Uppsala University, Uppsala, Sweden

**Keywords:** Alcohol use, Substance use, Psychiatry, Screening, Brief intervention, Obstacles

## Abstract

**Objective:**

In Sweden, national guidelines recommend that all staff in the healthcare system systematically screen patients for alcohol use and illicit substance use. Where hazardous use is identified, it should be addressed as soon as possible, preferably through brief interventions (BI). Results from a previous national survey showed that most clinic directors stated that they had clear guidelines for screening alcohol use and illicit substance use, but that fewer staff than expected used screening in their work. This study aims to identify obstacles and solutions to screening and brief intervention, based on survey respondents’ free-text responses to open-ended questions.

**Results:**

A qualitative content analysis yielded four codes: guidelines, continuing education, cooperation and resources. The codes indicated that staff would need (a) clearer routines in order to optimize compliance with the national guidelines; (b) more knowledge about how to treat patients with problematic substance use; (c) better cooperation between addiction care and psychiatry; and (d) increased resources to improve routines at their own clinic. We conclude that increased resources could contribute to better routines and cooperation, and provide increased opportunities for continuing education. This could increase guideline compliance and increase healthy behavior changes among patients in psychiatry with problematic substance use.

**Supplementary Information:**

The online version contains supplementary material available at 10.1186/s13104-023-06389-w.

## Introduction

Although up to 30% of patients in psychiatry are known to hazardously or harmfully use alcohol and drugs, it has been a challenge for psychiatry to identify and manage such problematic use within routine care [1]. Previous research on barriers and facilitators for screening and treating substance use disorders in psychiatry has shown that staff turnover is a barrier, in addition to limitations in number and length of treatment sessions or low internal motivation of the patient [2]. In primary care, lack of clinical knowledge and training, lack of clinical space, as well as time pressure have been reported as barriers to addressing substance use [3]. In-depth knowledge is lacking about barriers for treatment of individuals with co-occurring disorders, although an integrative literature review identified obstacles including both patient characteristics and structural barriers [4, 5].

A national effort in Sweden to survey psychiatry clinic directors and staff explored routines for identifying and managing problematic alcohol use and illicit substance use among patients, yielding a broad investigation of how barriers and facilitators were perceived, The main finding showed a considerable gap between national guidelines for managing substance use and actual psychiatric practice [6]. This research note reports a qualitative analysis of free-text comments from the survey, to add depth to current understanding of how to improve clinical management of problematic substance use among patients in psychiatry.

The original survey outcomes showed that most clinic directors reported guidelines to screen for alcohol (93.1%) and illicit substance use (78.9%) Some clinic directors also reported that they had guidelines for delivering brief interventions when identifying hazardous alcohol use (50%) and hazardous illicit substance use (35.9%) [6]. Discouragingly, fewer staff reported screening for alcohol (66.6%) and illicit substance use (57.8%), and only about a third of staff reported that they used brief interventions when identifying hazardous alcohol use (36.7%); however, the low rate of brief intervention for hazardous illicit substance use that staff reported (35.7%) was in parity with clinic directors’ reported guidelines. Additional analysis of the survey findings showed that staff who were trained in screening patients for alcohol or illicit substance use were more likely to actually screen patients than staff who had not received any training [2].

Open-ended questions in the national surveys [2] allowed respondents to complement their fixed responses with free text, and these data have not previously been reported. This research note presents findings from a qualitative analysis of these free-text responses.

## Methods

The study was conducted by obtaining the e-mail addresses of all clinic directors in outpatient psychiatry in the 21 healthcare regions in Sweden. An email with information on the study purpose, with a link to the online survey, was sent out to 228 managers and followed by two reminders to non-respondents. One of the questions in the survey asked if the clinic directors were willing to forward a link to a second survey to their clinical staff. If so, directors were also asked to indicate the number of employees to whom they would forward the survey and the occupations of their staff; thereafter they were sent a new survey link for distribution to their employees, estimated at about 1230. Two reminders were sent to the clinic directors to encourage their staff to answer. Data were collected from two separate cross-sectional online surveys, one for 132 managers in outpatient psychiatric clinics and the other for 522 staff. Table 1 shows charecteristics of participants. The survey for clinic directors included 18 questions, 5 with free-text options. The staff survey included 38 questions, of which 8 had an additional free-text response option, ending with an optional free-text question where participants could add any further thoughts on the issue of substance use among patients in psychiatry. Both surveys were disseminated via SurveyXact, a web survey tool. Complete survey texts are available in the survey report under Additional material.

[6]. See Supplementary materials A1 and A2 for the questions eliciting the free-text responses analyzed here.

Before the original national survey was sent out, an ethics review was conducted, which resulted in a consultative statement (ref. no. 2012/1695-31/5) by the Regional Ethics Review Board in Stockholm, Sweden, stating that no ethical review was considered necessary as no personal health data were collected from respondents.

**Table 1:** Characteristics of clinic directors and staff who responded to the survey

Characteristics		Clinic Directors (n = 132)		Staff (n = 522)	
Age /Mean (SD)		54.0 (8.2)		48.5 (10.8)	
Gender/Type of clinic, %, n		%	n	%	n
Gender	Male	22.7	30/132	22.8	119/520
Type of clinic	General	58.3	77/130	56.7	295/520
	Specialist*	26.9	35/130	20.5	107/520
	Psychosis	13.8	18/130	22.6	118/520

* In this category we included all specialist clinics other than psychosis (bipolar disorder/obsessive compulsive disorder (OCD)/neuropsychiatric clinics etc.)

The qualitative analysis reported here elicited the latent content of the free text responses from two surveys [6], consisting of 167 free-text responses from the clinic director survey and 477 free-text responses from the staff survey. The analysis was conducted manually, using paper printouts. Free-text responses for managers and staff were analyzed together since they did not differ in themes. Content analysis was applied, allowing themes to emerge from the text based on the researchers’ interpretation. The researchers did not define any themes in advance [7].

To achieve credibility in the study, the method is described in detail. The analysis was conducted in four steps. Step one: a careful reading of all free text responses, yielding an overall impression of the content. Step two: meaning units consisting of one or more sentences or paragraphs were selected. Step three: the underlying meanings were condensed to prepare for the fourth step, where the condensations were interpreted and expressed in terms of themes. Table 2 shows an example of the progression.

**Table 2:** Example of the structural analysis from meaning units to themes

Meaning unit (step 2)	Condensed meaning unit (3)	Theme (4)
Better adherence to guidelines. Addiction perspective is sometimes forgotten.	Better adherence to guidelines.	Guidelines
Continuous skills enhancement regarding new drugs and psychosocial treatment models is always important to retain expertise.	Continuous skills enhancement is important to retain expertise.	Continuing education
Believing that addiction care and psychiatry to a much greater extent should cooperate in their contacts with dependent patients.	Addiction care and psychiatry should cooperate.	Cooperation
Increased resources for even more improvement. Have already good cooperation with the municipality.	Increased resources for improvement.	Resources

### Naïve understanding

Based on the authors’ prior knowledge of the subject, topics expected to emerge during the analysis were lack of time and the need for education. During the first readings, additional topics were identified: a lack of systematicity in working methods, a lack of knowledge, a need for further training, a lack of resources at the clinics as well as a need for improved cooperation with other caregivers.

## Results

The qualitative analysis elaborated on the original survey finding concerning the **gap between national guidelines and daily practice**. Respondents described that they lacked clear guidance and routines to facilitate their work in encountering patients with problematic substance use. Staff members would prefer a better-defined structure regarding how to apply guidelines for addressing substance use, and how to increase guideline adherence, to help staff remember to maintain the addiction perspective. By following existing clinic guidelines, clinical work could become more efficient and contribute to patients’ receiving equal quality of care. However, there was a concern that compliance with the guidelines would create additional work in an already strained organization.

*“It is clear to me when I respond to the questionnaire that procedures need to be clarified and followed up.“ Survey for clinic directors (no. 122).*

*“Clearer structure [needed] to make sure that we don´t miss any patients with problems.” Survey for clinic directors (no.121)*

The free-text responses also showed **a desire and a need for increased knowledge and continuing education**. Routines and tools already available, such as screening and personalized feedback, or various forms of digital intervention, need to be prioritized in the workday agenda, but available tools are not now sufficiently used. More education about addiction and its harmful effects was desired as well as more time for reflection and exchange of experiences. Respondents also needed continuous education, not just ad hoc projects. Continuing to self-educate oneself or learn via courses offered through work was perceived as a path to develop professionally, but requires continuous support.

*“I´m surprised at how little they know in psychiatry about this. I worked about 12 years in addiction care, which means that I’m pretty proficient in this. The knowledge gap is large and a bit surprising when it previously has been integrated as part of psychiatry. Specialization is good but at the same time the holistic approach to the human being is lost.“ Staff survey (no. 201)*

*“Continuous updating or education regarding drugs on an annual basis via interactive web training created by the addiction care”. Staff survey (no.9)*

Several clinics **cooperated with addiction care**, but the majority lacked this or had a cooperation that needed to be improved. In this context, the need to enhance the overall organization was mentioned so that work at the local level could be improved for patients with co-occurring disorders. A lack of cooperation had a negative effect on both staff and patients, and respondents hoped for better cooperation in the future, important both for the patient’s benefit but also for the staff to save time and energy.

*“I consider that addiction treatment and psychiatric care should cooperate to a much greater extent in managing dependent patients.“ Staff survey (no. 59)*

*“Team or working in a network is a success factor when working with people with complex care and support needs.” Staff survey (no.395)*

A **lack of resources** was a final obstacle that emerged in the survey, meaning that both staff and time were lacking in order to best be able to address substance use among patients. Increasing staff density would reduce the burden on staff, patients would receive better care, and waiting times could be cut.

*“If I were to be really honest, more staff. With the cutbacks that psychiatric care has conducted for nearly two decades, we now have outstanding concerns. " Survey for clinic directors (no. 12)*

*“So, the biggest problem today is time and the availability of staff, and of course knowledge and methods.” Survey for clinic directors (no. 12)*

## Discussion

This qualitative analysis aimed to shed light on reasons for the gap between guidelines and practice concerning identification and management of problematic substance use in psychiatry. The findings have clarified in what way clinical staff in psychiatry do not fully adhere to existing guidelines and routines for addressing problematic substance use. Previous studies have shown that a lack of adherence to guidelines in the long run can lead to patients not being offered the right care, where the care can be directly harmful or ineffective [8].

Experiencing low compliance to guidelines was described by the respondents as problematic. Implementation of evidence-based treatment methods can be facilitated by developing a better understanding of how new methods can affect efficiency and also by developing an understanding of what actions need to be taken and what they will lead to [9]. Staff expressed a desire to receive continuing education, above all to learn more about psychiatry and addictive disorders. To meet the need for knowledge development, formal continuing education could lead to continuous development of clinical competencies [10]. Learning could take several forms, for example more informal learning in a slow process where the individual continues to learn at the same time as the activity continues, so new lessons are consolidated in practice [11] – or in lifelong learning [12].

The tasks that staff prioritize and the type of treatment selected for the patient can also be affected by factors that are not controllable, such as time pressure and lack of resources [13]. Stressful circumstances create an increased risk that staff make incorrect assessments and ensuing disadvantageous treatment decisions for the patient [14]. This means that the lack of resources can seriously endanger patient security.

This study strengthens findings from previous studies mentioned in this article and the next step could be a study that explores which resources need to be prioritized in order to contribute to increased screening and treatment to achieve better care for patients with addictive disorders.

## Conclusion

Healthcare cuts over the past two decades in Sweden have left their mark and it is difficult to generate new resources. This study suggests that increased resources and training for the staff could enable them to have time to develop more cooperation between the clinics and lead to competence development, including formal training in addiction and training on current guidelines and routines. This could increase guideline adherence and eventually reduce the workload, thus saving resources.

## Limitations

Due to the nature of the cross-sectional survey, it was not possible to ask supplementary follow-up questions, which in some cases would have been desirable. Since the data were obtained through an online survey, some findings may have been missed as they were recorded by the respondents themselves, perhaps meaning that the raw data were more concise than they would have been if the interview had been conducted orally.

## Electronic supplementary material

Below is the link to the electronic supplementary material.

Supplementary Material 1

## Data Availability

The data sets from the qualitative analysis are available from the corresponding author after reasonable request.
