# Supplementary material for: Screening and treating problematic substance use among patients in psychiatry – obstacles and solutions
Source: BMC Res Notes. 2023 Jun 22;16:113. doi: 10.1186/s13104-023-06389-w (PMC10288691; doi:10.1186/s13104-023-06389-w)
Supplement: Supplementary file 1 — Supplementary Material 1 [file 13104_2023_6389_MOESM1_ESM.pdf]

Supplementary material A1

Survey to clinic directors of psychiatric outpatient clinics in Sweden – English version

Questions with free-text responses

7. Do clinicians at your clinic have clear guidelines to take any specific actions when a patient has hazardous alcohol use?
(1) Yes, brief intervention
(2) Yes, other_______
(3) Yes, referral to addiction care, social services or primary care
(4) No, we have no guidelines to take any action
(5) Uncertain / do not know

8. Do clinicians at your clinic have clear guidelines take any specific action when a patient has alcohol abuse or dependence?
(1) Yes, brief intervention (screening and feedback, alcohol diary, identifying risk situations etc)
(2) Yes, other_______
(3) Yes, referral to addiction care, social services or primary care
(4) No, we have no guidelines to take any action
(5) Uncertain / do not know

10. Do clinicians at your clinic have clear guidelines take any specific action when a patient has a hazardous illicit substance use?
(1) Yes, brief intervention (screening and feedback, alcohol diary, identifying risk situations etc)
(2) Yes, other_______
(3) Yes, referral to addiction care, social services or primary care
(4) No, we have no guidelines to take any action
(5) Uncertain / do not know

11. Do clinicians at your clinic have clear guidelines to take any specific action when a patient has illicit substance abuse or dependence?
(1) Yes, brief intervention (screening and feedback, alcohol diary, identifying risk situations etc)
(2) Yes, other_______
(3) Yes, referral to addiction care, social services or primary care
(4) No, we have no guidelines to take any action
(5) Uncertain / do not know

14. When it comes to identification and management of patients with alcohol and/or illicit substance problems in psychiatry, what do you think could be improved in the work of your clinic? You can note more than one alternative.
(1) Better contact with addiction care
(2) More education to healthcare professionals about alcohol and illicit substances
(3) More information for patients
(4) Other: __________
(5) Nothing, everything works optimally

Full survey text available in Additional material at:
Sundström C, Petersén E, Sinadinovic K, Gustafsson P, Berman AH. Identification and management of alcohol use and illicit substance use in outpatient psychiatric clinics in Sweden: a national survey of clinic directors and staff. Addiction science & clinical practice. 2019;14(1):10. *DOI: 10.1186/s13722-019-0140-x*

Supplementary material A2

Survey to staff members in psychiatric outpatient clinics in Sweden – English version

Questions with free-text responses

13. What actions do you usually take if you notice that a patient has a hazardous alcohol use? By hazardous use, we mean alcohol use at a level that carries an increased risk of harmful physical, mental and social consequences, but which cannot be said to be abuse or dependence. You can enter several options.
(1) Provide brief intervention (eg screening and feedback, alcohol diary, identification of risk situations, etc.)
(2) Provide information on the negative effects of alcohol
(3) Try to open a dialogue with the patient about alcohol habits
(4) Offer contact with other staff at clinic
(5) Offer referral to other healthcare providers, eg addiction care
(6) I do not take any action
(7) Other, what: ____

14. What actions do you usually take if you notice that a patient has an alcohol abuse or dependence? By abuse or dependence we mean a use of alcohol that causes harmful consequences and recurring problems in relation to the environment. You can enter several options.
(1) Provide brief intervention (eg screening and feedback, alcohol diary, identification of risk situations, etc.)
(2) Provide information on the negative effects of alcohol
(3) Try to open a dialogue with the patient about alcohol habits
(4) Offer contact with other staff at clinic
(5) Offer referral to other healthcare providers, eg addiction care
(6) I do not take any action
(7) Other, what: ____

18. (If yes on Q17: Has it ever happened that you have chosen not to raise the issue of alcohol habits during a treatment even though you thought) Why did this happen? You can fill in several options.
(1) I don't have enough time
(2) I do not have enough knowledge about alcohol problems
(3) The patient is not interested in discussing alcohol habits
(4) The patient already has an established contact with other healthcare providers, such as addiction care or social services
(5) Other, state what: ____

Full survey text available in Additional material at:
Sundström C, Petersén E, Sinadinovic K, Gustafsson P, Berman AH. Identification and management of alcohol use and illicit substance use in outpatient psychiatric clinics in Sweden: a national survey of clinic directors and staff. Addiction science & clinical practice. 2019;14(1):10. *DOI: 10.1186/s13722-019-0140-x*

24. What actions do you usually take if you notice that a patient has hazardous illicit substance use? By hazardous use, we mean use that carries an increased risk of harmful physical, mental and social consequences, but cannot be said to be abuse or dependence. You can fill in several options.
(1) Provide brief intervention (eg screening and feedback, illicit substance diary, identification of risk situations, etc.)
(2) Provide information on negative effects of illicit substance use
(3) Try to initiate a dialogue with the patient on illicit substance use
(4) Offer contact with other staff on reception
(5) Offer referral to other healthcare providers, eg addiction care or social services
(6) I do not take any action
(7) Other, what: ____

25. What actions do you usually take if you notice that a patient has illicit substance abuse or dependence? By illicit substance abuse or dependence we mean an intensive use of illicit substances that causes harmful consequences and recurring problems in relation to the environment. You can fill in several options.
(1) Provide brief intervention (screening and feedback, illicit substance diary, identification of risk situations etc.)
(2) Provide information on negative effects of illicit substance use
(3) Try to initiate a dialogue with the patient on illicit substances / illicit substance habits.
(4) Offer contact with other staff at the reception.
(5) Offer referral to other healthcare providers, eg addiction care or social services
(6) I do not take any action
(7) Other, what: ____

29. Why did it happen? You can fill in several options.
(1) I don't have the time
(2) As a clinician, I do not have enough knowledge about illicit substance use
(3) The patient is not interested in discussing illicit substance use
(4) The patient already has an established contact with addiction care/social services
(5) Other, what: ____

34. When it comes to identification and management of patients with alcohol and illicit substance use problems in psychiatry, what do you think could be improved in the work at your clinic? You can enter several options.
(1) More training to staff on mental and somatic consequences of alcohol and illicit substances
(2) More training for staff on therapy methods in substance abuse.
(3) Improved contact with addiction or social services.
(4) Written information material to offer patients
(5) Clearer management decisions on how to deal with problematic use of alcohol / illicit substances
(6) Nothing, the clinic works satisfactorily in this area
(7) Other, what: ____

35. If you have additional views on problematic use of alcohol/illicit substances among patients in psychiatry we are grateful if you share them below
.____
____
____

Full survey text available in Additional material at:
Sundström C, Petersén E, Sinadinovic K, Gustafsson P, Berman AH. Identification and management of alcohol use and illicit substance use in outpatient psychiatric clinics in Sweden: a national survey of clinic directors and staff. Addiction science & clinical practice. 2019;14(1):10. *DOI: 10.1186/s13722-019-0140-x*
